# Modelling Improvements in Cell Yield of Banked Umbilical Cord Blood and the Impact on Availability of Donor Units for Transplantation into Adults

**DOI:** 10.1155/2013/124834

**Published:** 2013-02-20

**Authors:** Natasha Kekre, Jennifer Philippe, Ranjeeta Mallick, Susan Smith, David Allan

**Affiliations:** ^1^Blood and Marrow Transplant Program, Division of Hematology, Department of Medicine, University of Ottawa, 501 Smyth Rd., Box 704, Ottawa, ON, Canada K1H 8L6; ^2^OneMatch Stem Cell and Marrow Network, Canadian Blood Services, 40 Concourse Gate, Ottawa, ON, Canada K2E 8A6; ^3^Clinical Epidemiology Program, Ottawa Hospital Research Institute, 501 Smyth Rd., Ottawa, ON, Canada K1H 8L6; ^4^Regenerative Medicine Program, Ottawa Hospital Research Institute, 501 Smyth Rd., Ottawa, ON, Canada K1H 8L6

## Abstract

Umbilical cord blood (UCB) is used increasingly in allogeneic transplantation. The size of units remains limiting, especially for adult recipients. Whether modest improvements in the yield of cells surviving storage and thawing allow more patients to proceed to transplant was examined. The impact of improved cell yield on the number of available UCB units was simulated using 21 consecutive anonymous searches. The number of suitable UCB units was calculated based on hypothetical recipient weight of 50 kg, 70 kg, and 90 kg and was repeated for a 10%, 20%, and 30% increase in the fraction of cells surviving storage. Increasing the percentage of cells that survive storage by 30% lowered the threshold of cells needed to achieve similar engraftment rates and increased numbers of UCB units available for patients weighing 50 (*P* = 0.011), 70 (*P* = 0.014), and 90 kg (*P* = 0.003), controlling for differences in HLA compatibility. Moreover, if recipients were 90 kg, 12 out of 21 patients had access to at least one UCB unit that met standard criteria, which increased to 19 out of 21 patients (*P* = 0.035) when the fraction of cells surviving storage and thawing increased by 30%. Modest increases in the yield of cells in banked UCB units can significantly increase donor options for adult patients undergoing HSCT.

## 1. Introduction

Umbilical cord blood (UCB) is used increasingly as an alternative source of hematopoietic stem cells for allogeneic transplantation into pediatric and adult patients. The National Marrow Donor Program recently reported that 10% of adult hematopoietic stem cell transplants (HSCT) and 41% of pediatric transplants utilized UCB as the graft source [1]. There are many reasons for the increased utility of UCB. Firstly, less than 30 percent of potential HSCT recipients will have a matched related donor available [2] and the trend towards increased reliance on alternative donor sources will continue well into the future based on reduced fertility rates observed in recent decades [3]. This has led to increased utilization of unrelated sources of stem cell grafts, including widespread public cord blood banking. UCB transplantation requires less stringent HLA matching compared with bone marrow or peripheral blood stem cell grafts [4] and thereby helps to overcome barriers to donor availability for many patients, and especially patients from ethnic minorities [5].

 The size of many adult patients often limits the availability of cord blood units due to the reduced number of cells per kilogram of recipient body weight, or the effective stem cell dose. Low cell doses may preclude transplantation or contribute to delayed engraftment [6], transplant-related mortality, and survival [7, 8]. Combining more than one UCB unit for transplantation into adults has been one approach to overcome limiting cell doses [9, 10], but this approach substantially increases costs of transplantation, making it unaffordable in many jurisdictions. Measurement of postthaw cell number, viability, and function have highlighted the significant impact of processing, cryopreservation, and thawing on cell number and function. Reports have suggested that the recovery of total nucleated cells (TNC) prior to infusion at time of transplant ranges from 50 to 80 percent [11–16]. This further limits the applicability of UCB transplantation for adult patients. Thresholds of prestorage cell numbers have been established for timely engraftment of adult recipients of UCB transplantation. Total nucleated cell dose thresholds depend on the degree of HLA disparity, with a greater TNC dose required for each additional HLA mismatch [17, 18].

 Availability of donor units of sufficient size continues to be a limiting factor for many patients, especially as requests for available backup units become more common in case of graft failure [17]. Research to address improvements in cell recovery through optimization of cryopreservation conditions is ongoing [19–21]. Many studies have addressed potential cell expansion methods although this remains an area of active research and will likely be associated with significant cost [22]. In this study, we sought to quantitatively model the required improvements in TNC yield that could improve access to UCB donors for adult-sized patients undergoing HSCT. Our approach uses anonymized actual searches and simulates the impact of improved cell yield following storage and thawing on the number of available cord blood units. The goal of our study was to provide an estimate of the improvement in cell yield required to impact the pool of available donors for adult patients that could influence cord blood banking practices by blood establishments.

## 2. Materials and Methods

### 2.1. Match Selection

Twenty-one consecutive preliminary umbilical cord blood searches from the OneMatch Stem Cell Network of Canadian Blood Services were anonymized and obtained for use in the modeling analysis. In our study, we used results from the Cord Blood Match Program of the Bone Marrow Donors Worldwide (BMDW) for actual preliminary searches performed by Canadian Blood Services. The BMDW search program provides preliminary results from the inventories of 46 cord blood banks in 30 countries with a total combined inventory in excess of 500,000 units. Canadian Blood Services routinely uses the Cord Blood Match Program of BMDW to obtain preliminary search results from cord blood bank inventories. The research protocol was approved by the research ethics boards at The Ottawa Hospital Research Institute and Canadian Blood Services. 

### 2.2. Modelling the Impact of Improved Cell Yield on Cord Blood Search Results

A model was created based on current published criteria used for selection of umbilical cord blood units for HSCT [18]. These criteria incorporate the degree of HLA-A, HLA-B, and HLA-DR compatibility with thresholds of TNC. A TNC dose of 1.5 × 10^7^/kg was used to identify 6/6 HLA-matched units, 2.5 × 10^7^/kg was used to identify 5/6 HLA-matched units, and 5.0 × 10^7^/kg was used to identify 4/6 HLA-matched units. We calculated the TNC dose per kg in each search using 50 kg, 70 kg, and 90 kg to simulate the range of weights typical for adult transplant recipients. The total number of units available, including HLA-matched and HLA-mismatched units, was determined at each weight for each search. We repeated the calculation and assumed an increase of 10%, 20%, or 30% in the percentage of cells that survived storage and thawing to determine the number of available cord blood units that exceeded the minimum criteria for each of the recipient weight categories. The hypothetical increase in percentage of cells that survived the storage and thawing process allowed us to calculate a lowered effective TNC threshold that we used for identifying units that could be selected for transplantation (see Table 1). Importantly, even a 30% increase in the fraction of cells surviving over the standard level of 70% would yield an achievable 91% of cells surviving storage and thawing. As an example,

 standard TNC threshold at collection = yield after thaw/(% cells surviving).

If we assume an increase of 10% in the fraction of cells surviving storage, we can calculate a new lower threshold for identifying units associated with the same likelihood of timely engraftment (i.e., units with the same yield of cells after thawing) as follows:

- new TNC threshold at collection = yield after thaw/(% cells surviving × 1.1), or
- new TNC threshold at collection = standard TNC threshold/1.1.

### 2.3. Statistical Analysis

A mixed effects model was used to determine the impact of two variables: (1) degree of HLA matching and (2) fraction of cells surviving storage and thawing, on the mean number of umbilical cord blood units available. In cases of statistical significance, multiple comparisons were performed to compare groups within the variable using Tukey's adjustment. Mean values were reported ±1 standard error unless otherwise stated. Proportions were compared using chi-squared analysis.

## 3. Results

**Figure 1:**
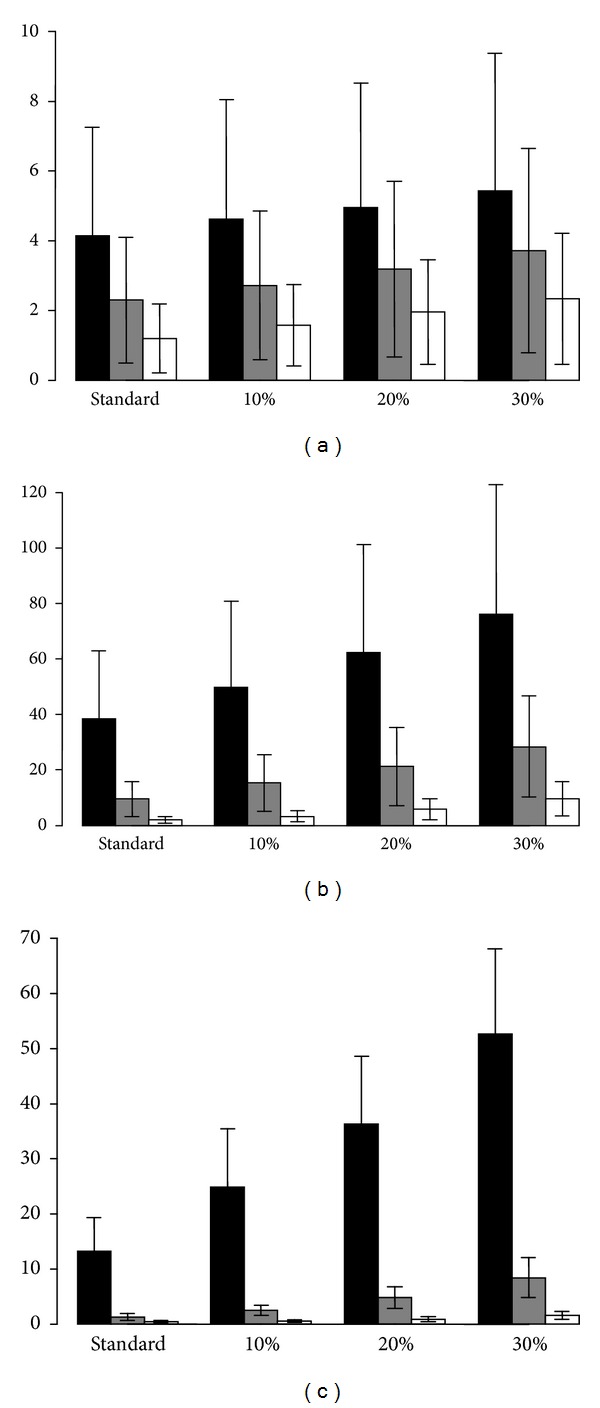
Mean number of available UCB units with increased yield of cells surviving storage and thawing. Mean number of UCB units identified if patients weighed 50 kg (black), 70 kg (grey), and 90 kg (white). Mean number of units that were 6/6 HLA matched (a), 5/6 HLA matched (b), and 4/6 HLA-matched (c) are presented using the standard threshold criteria for unit selection (standard) and if there was a 10%, 20%, or 30% increase in the fraction of cells surviving storage and thawing. Error bars represent the standard error of the mean.

An increase in the percentage of cells that survive the storage and thawing process lowered the effective threshold of TNC needed at the time of collection and demonstrated a trend towards increased mean number of available UCB units for patients weighing 50, 70, and 90 kg at all levels of HLA compatibility (see Table 2). A mixed effects model was used to assess the impact of increasing the percentage of surviving cells by 10%, 20%, and 30% and the effect of HLA-matching (6/6 versus 5/6 versus 4/6) on the mean number of umbilical cord units available per patient. Both variables were significantly associated with increased numbers of available donors (*P* = 0.01 for the increase in the fraction of cells that survive storage and thawing and *P* < 0.0001 for degree of HLA match). Multiple comparisons revealed that a 20% (*P* = 0.047) and 30% (*P* = 0.0015) improvement in the fraction of cells surviving storage and thawing for a 50 kg patient increased the mean number of UCB units available from 21.4 (standard conditions) to 37.0 (20% improvement) and 47.1 (30% improvement), respectively. For a weight of 70 kg and 90 kg, a 30% increase in the fraction of cells surviving storage and thawing (*P* = 0.0021 and *P* = 0.0005, resp.) was associated with a significant increase in the number of available units (5.1 to 14.3 (for 70 kg patients) and 1.3 to 4.7 (for 90 kg patients), resp.) (see Table 2). The number of donor units that were HLA matched or mismatched (5/6 and 4/6 matches) for patients weighing 50, 70, and 90 kg is presented in Figures 1(a)–1(c) for standard conditions and for theoretical increases of 10%, 20%, and 30% in the survival of cells surviving storage and thawing.

To better appreciate the clinical impact of improving the yield of cells that survive storage, we determined the number of patients who had at least one donor available using standard selection criteria and then determined the number of patients with available donors using the new effective TNC thresholds based on 10%, 20%, and 30% improvement in percentage of cells that survive storage and thawing. If all patients weighed 50 kg, all patients in the study would have at least one available donor that met standard criteria. If all patients weighed 70 kg, only 19 of the 21 patients had an available unit identified using standard selection criteria; however, with a 30% increase in the fraction of cells surviving, the reduced effective TNC dose allowed all patients to have an available UCB unit. Most strikingly, if all patients weighed 90 kg, just 12 of 21 patients had an available unit and this increased to 19 of 21 patients (*P* = 0.035) when the fraction of cells surviving storage and thawing increased by 30%.

To gain insight regarding the applicability of our results on a broader scale, we modelled the number of searches needed to appreciate clinically significant changes in the number of 6/6 HLA-matched donor units that would be available for adult patients weighing 50, 70, and 90 kg in the setting of a 10% improvement in the percentage of cells that survive storage and thawing. We chose this modest level of improvement in the yield of cells to highlight the potential impact that could be realized from minimal improvement in cell yield. We determined the sample size needed to achieve 90% power at 0.05 level of significance and extrapolated the results of our study using the standard deviation and mean number of HLA-matched donor units identified in the actual searches for hypothetical donors weighing 50, 70, and 90 kg. Using this approach, we determined that cord blood searches for only 37, 78, and 60 transplant recipients would be required for patients weighing 50, 70, and 90 kg, respectively, to identify a significant increase in the number of available donor units if the yield of cells that survived processing and storage increased by just 10%. That is to say, studies designed to detect a significant increase in the availability of HLA-matched cord blood units for adult patients that would be associated with a 10% increase in the yield of cells would need to enrol between 37 and 78 adult patients, depending on the weight of the patients.

## 4. Discussion

In this study, we have demonstrated the marked impact on the availability of banked cord blood units that would become available for transplantation into adult patients if the yield of surviving cells in banked UCB units could increase by up to 30%. Notably, our simulated modeling reveals that more patients would have at least one donor unit available if the process of storage and thawing could be improved by 30%. If the trends we observed in this small study are applied to a larger sample size, we expect that improvements of just 10% could significantly improve donor options on a national and international scale. The results of our research are most notable for a patient weight of 90 kg which reflects an increasing proportion of the adult population. Our findings strongly support the need for improvements in storage, cryopreservation, and thawing techniques that could yield even modest benefits in the final yield of cells in thawed units.

To the best of our knowledge, our study is the first to quantify the magnitude of improvement in the yield of cells that survive storage and thawing that is needed to improve access to donors for adult patients who are candidates for cord blood transplantation. The dose of total nucleated cells remains the chief determinant that impacts on the selection of umbilical cord blood units [17, 23, 24]. Recent research suggests that a TNC count of 1.5 × 10^9^ in collected units is associated with a marked increase in likelihood of selection by transplant centres. Many cord blood banks have increased the threshold for collection to maximize the percentage of their inventory represented by larger units that meet these criteria. This strategy, however, means that as many as 90% of collected units are not suitable for banking due to insufficient cell numbers and introduces challenges for cord blood bank viability. It is possible, however, that greater understanding of factors associated with high TNC counts could improve the efficiency associated with identifying and banking units with high cell counts. Another approach to overcoming limiting doses of cells in banked units that have been embraced by some transplant centres is a strategy of combining cord blood units to reach specific cutoffs for total nucleated cells per kg. This approach places greater strain on the resources of cord blood banking establishments as the inventory will be depleted more quickly and the high fees for retrieving banked units introduce significant financial barriers for transplant centres and health care authorities that are prohibitive in many jurisdictions. Strategies to improve the yield of cells that can survive the processing, storage, and thawing steps are less studied and could be cost-effective. Carbohydrate-based inhibitors of ice recrystallization [25], including antifreeze glycoproteins [26], are able to reduce the amount of dimethylsulfoxide required for safe storage of umbilical cord blood and appear promising as a means of improving the yield of viable cells. Other methods of improving the yield of cells include lyophilisation of cells [27] and novel storage methods. In addition, volume reduction techniques remain inefficient, particularly for large cellular units, and improvements in volume reduction may confer benefits on the yield of available cells [28, 29]. A final consideration for overcoming the limited cell dose in stored cord blood units is expansion of the stem and progenitor cell populations. Many groups have considered this approach using ex vivo cytokines, HOXB4, and other key regulators of hematopoietic stem cells [22, 30]. Cell expansion methods, however, will likely be resource intensive and will require specialized laboratories that will be subject to additional regulatory oversight which may limit widespread applicability.

One limitation of the current study is the sample size. Although we detected significant differences in the availability of donors using a model that increased the yield of cells by 30% when considering patients weighing 90 kg, we believe that smaller improvements in the fraction of cells that survive storage and thawing will have incremental effects on the availability for donors of patients of all weights. Extrapolation of our results suggests studies enrolling as few as 80 patients would be sufficiently powered to detect significant differences in the availability of HLA-matched cord blood units for adult patients associated with as little as a 10% increase in the fraction of cells that survive storage and thawing. The precise relationship between the degree of improvement in cell yield and impact on the availability of cord blood units for transplantation remains unproven for patients less than 50 kg. Moreover, it is important to acknowledge the evolving landscape in cell enumeration and that variable approaches to the standardization of TNC and CD34 enumeration may have impacted our results. Finally, we acknowledge that evolving practices by cord blood banks may allow one to identify particular banks that embrace practices associated with the highest quality processing and storage techniques. It would be interesting to examine the impact of improved cellular yields in the context of units derived only from banks embracing practices associated with the highest quality standards. 

In conclusion, our study provides a clear demonstration that realistic and achievable improvements in the yield of cells surviving storage and thawing can have a profound impact on the availability of cord blood units for transplantation into adults recipients. Research that demonstrates modest improvements in the yield of cells in banked cord blood units could have an important effect on the field of cord blood banking and transplantation on a global scale.

## Figures and Tables

**Table 1:** New TNC thresholds if percentage of cells surviving storage and thawing increased by 10%, 20%, and 30%.

Thresholds × 10^7^/kg	6/6 HLA match	5/6 HLA match	4/6 HLA match
Standard threshold	1.5	2.5	5.0
New threshold (10%)	1.36	2.27	4.55
New threshold (20%)	1.25	2.08	4.17
New threshold (30%)	1.15	1.92	3.85

**Table 2:** Mixed effects model on the impact of increasing the fraction of cells surviving storage and thawing compared to standard yields and the impact of HLA compatibility on the number of available donor units (least square means reported).

	Available donor units for patient weight		
	50 Kg	70 kg	90 kg
Percentage of cells surviving^a^			
Standard	21.3	5.1	1.3
110% (*P* versus standard)	29.0 (0.33)	7.6 (0.38)	1.9 (0.50)
120% (*P* versus standard)	37.0 (0.047)	10.5 (0.061)	3.0 (0.068)
130% (*P* versus standard)	47.1 (0.002)	14.3 (0.002)	4.7 (0.0005)
Degree of HLA-match^b^			
6/6 HLA matched	4.8	3.0	1.8
5/6 (*P* versus 6/6)	56.6 (0.016)	18.6 (<0.0001)	5.2 (<0.0001)
4/6 (*P* versus 6/6)	39.4 (<0.0001)	6.5 (0.17)	1.3 (0.58)
4/6 (*P* versus 5/6)	39.4 (<0.0001)	6.5 (<0.0001)	1.3 (<0.0001)

^a^ *P* values for mixed effects model considering increases in yield of cells surviving storage and thawing (*P* = 0.011 for patient weight of 50 kg, 0.014 for 70 kg, and 0.003 for 90 kg).

^b^ *P* values for mixed effects model considering degree of HLA match (*P* < 0.0001 for patient weight of 50 kg, 70 kg, and 90 kg).
